# Quality of life and satisfaction with life of malaria patients in context of acceptance of the disease: quantitative studies

**DOI:** 10.1186/1475-2875-11-171

**Published:** 2012-06-29

**Authors:** Katarzyna Van Damme-Ostapowicz, Elżbieta Krajewska-Kułak, Emilia Rozwadowska, Wacław L Nahorski, Romuald Olszański

**Affiliations:** 1 Department of Integrated Medical Care, Medical University of Białystok, Białystok, Poland; 2 Interdisciplinary Institute of Marine and Tropical Medicine, Medical University of Gdańsk, Gdańsk, Poland; 3 Department of Maritime and Tropical Medicine, Military Medical Institute, Gdynia, Poland

**Keywords:** Malaria, Quality of life, Satisfaction with life, Acceptance of the disease

## Abstract

**Background:**

Health status is one of the basic factors of a high quality of life and the problem of the acceptance of illness is important for adaptation to the limitations imposed by it. The purpose of the study was the evaluation of the quality of life, satisfaction with life and the acceptance of illness by malaria patients, as well as the discovery of a relationship between studied parameters.

**Methods:**

The study was undertaken in August 2010, on 120 Nigerian patients with confirmed malaria. A method of diagnostic survey, based on standardized scales - Acceptance of Illness Scale, The Satisfaction With Life Scale and a standardized survey questionnaire World Health Organization Quality of Life/BREF - was used in this study. Descriptive statistics, variability range, 95% confidence interval, correlation analysis, Spearman’s non-parametric correlation coefficient, Mann–Whitney test and Kruskal-Wallis test were applied and the, so called, test statistics was calculated, followed by the calculation of the test probability *p*. Results of analyses were presented in a box graph, and a graph of dispersion.

**Results:**

A dominating share in the adjective scale of the AIS scale was the category of “no acceptance”, given by 71.7% of respondents. The average level of a “somatic domain” was 41.7, and of a “social domain” was 62.8. The mean satisfaction of life evaluation in the SWLS scale was 18 points. The correlation between acceptance of the disease and quality of life for the psychological domain was 0.39***, and between acceptance of the disease and satisfaction with life was 0.40***. The correlation between satisfaction with life and quality of life for the psychological domain was 0.65***, and between satisfaction with life and quality of life for the environment domain was 0.60***. The mean level of AIS for the studied population of men was 16.5, and test probability: *p* = 0.0014**, and for the environment domain the level was 50, and the test probability: *p* = 0.0073**. For quality of life in the social sphere the test probability: *p* = 0.0013** in relatively older individuals.

**Conclusion:**

The majority of people do not accept their condition. Evaluation of the quality of life was the highest in the social domain, and the lowest in the somatic domain. There is a statistically significant correlation between the level of acceptance of illness and the quality of life and satisfaction with life. The strongest correlation is found between satisfaction with life and the evaluation of the quality of life in psychological and environmental domains. Men evaluate their quality of life in the environmental domain higher and demonstrate a higher acceptance of their disease. There is a correlation regarding a significantly higher quality of life in the social sphere in relatively older people.

## Background

Malaria has been a major public health problem in Nigeria and many other sub-Saharan African countries [1,2]. During the past decade, malaria incidence and mortality rates have been cut in all regions of the world, according to the World Malaria Report 2011. In 2010, there were estimated 216 million cases of malaria in 106 endemic countries and territories in the world. An estimated 81% percent of these cases and 91% of deaths occurred in the WHO African Region. Globally, 86% of the victims were children under five years of age. There were an estimated 655,000 malaria deaths in 2010, which is 36,000 lower than the year before. While this 5% year-on-year decline represents significant progress, the mortality figures are still disconcertingly high for a disease that is entirely preventable and treatable [3].

Malaria constitutes a serious obstacle to the development of the human population and economy [4], and causes annual expenses of approximately eight billion euros in African countries [5]. Malaria influences the whole life of the affected population by an intensification of poverty, a limitation of education opportunities, and absenteism in schools and at work [5].

Studies on the quality of life of patients and healthy people, conducted by physicians and other health-related specialists, appeared in world literature as early as in the 1970s [6]. In medical sciences, studies on the quality of life may be regarded as a type of meta-analysis of current medical theories and practice, accepted diagnostic and therapeutic procedures, medical care and rehabilitation [7].

The concept of the quality of life is very useful for processes of health enhancement, therapy and holistic care, and for rehabilitation processes [8]. Researchers dealing with studies on the quality of life underline that the evaluation should consider the patient’s somatic condition, his/her mental status, social relations and physical fitness. For heath condition is one of the basic factors of high quality of life [9]. On the other hand, the level of acceptance of illness significantly influences adaptation to the limitations imposed by the disease, dependence on other people and a sense of own value. The above-mentioned determinants influence a subjective sense of the quality of life and determine the level of an individual’s own activity [10]. Each disease causes negative emotions, difficulties and forces to limit or make changes in social functions that one holds/performs [11].

Professional literature underlines the fact that the higher the level of disease acceptance is, the better the adaptation and the lower intensity of negative emotions in patients are [12]. Professional literature does not contain reports on the problem of the quality of life and the satisfaction with life of malaria patients in the context of acceptance of the disease. Therefore, conducting studies allowing the evaluation of the patients’ quality of life and general condition in relation to the discussed disease seem purposeful.

## Methods

### Study area and population

The study was carried out in August 2010 among 120 patients with confirmed malaria, diagnosed at the Madonna University Teaching Hospital in Elele, in South Nigeria, in the state of Rivers.

47% of subjects were women, and 53% men. Young (age below 30) and middle-aged (age below 50) people prevailed among the surveyed patients. The ratio of adolescents and elderly people was much lower, below 20% in each of those categories.

### Quantitative research methods

A method of diagnostic survey using two standardized scales: AIS and SWLS and a standardized survey questionnaire: WHOQoL-BREF were used for the achievement of objectives of the study.

The standardized AIS, exploring the patient’s acceptance of the disease, consists of eight questions describing the consequences of poor health condition. The questions relate to the limitations imposed by the disease, the lack of self-sufficiency, the sense of being dependent on others and the reduced sense of self-value. Each question contained a five-grade scale, and a surveyed participant determined his/her current health condition by marking one of the numbers: 1 – strongly agree, 2 – agree, 3 – do not know, 4 – disagree, 5 – strongly disagree.

A strong agreement means poor adaptation to the disease, and no agreement – acceptance of the disease. The sum of all the points, ranging between 8 and 40, is a measure of the level of acceptance. Three point ranges were created for the description of the level of acceptance. A score ranging from 8 to 18 stood for no acceptance to the disease, from 19 to 29 – a medium acceptance, and from 30 to 40 – a good acceptance.

The standardized SWLS is a measure of life satisfaction. Satisfaction with life is one factor in the more general construct of subjective well-being. Satisfaction with life can be assessed specific to a particular domain of life (e.g., work, family) or globally. The SWLS is a global measure of satisfaction with life. The SWLS consists of five items that are completed by the individual whose satisfaction of life is being measured. A surveyed participant “agrees” or “disagrees” with statements using a seven-grade scale of answers (“totally agree”, “agree”, “rather agree”, “neither agree nor disagree”, “rather disagree”, “disagree”, “totally disagree”). Answers are positively scored which means that the higher the score, the higher the satisfaction with life.

The standardized survey questionnaire *WHOQoL-BREF* brief version contains 26 questions. The instrument allows for the determination of the quality of life profile in four domains: physical domain, psychological domain, social relations and environment. Scores for those domains reflect the individual perception of the quality of life within the domains. The domain score is positive, which means that the higher score, the better the quality of life.

Moreover, the study used a patient survey questionnaire prepared especially for it, which was not validated, containing in its introductory part: information about the purpose of the study, the voluntary character and the anonymity of answers, information on the patient’s right to withdraw from the study at any stage and in any moment, and information on the method of filling in the questionnaire and of the scales. In the first part the survey questionnaire also contained five questions on the demographic data of the respondent (gender age, place of living, marital status, profession), and in the second part – 14 open questions regarding the health situation of the patient (medical history).

Survey questionnaires and scales were prepared in English, which is the official language in Nigeria. The filling in of questionnaires was performed in the presence of the Project Manager and of a student of the last year of the medical faculty at the Madonna University, who was trained in objectives and the assumptions of the study and who knew the local language. The study objectives and methods were also explained to each of the respondents before the start of the study.

### Statistical analysis

Statistical elaboration was performed using the STATISTICA software in the form of the following descriptive statistics: arithmetic mean, median, maximum and minimum value, standard deviation (*s*), centile 25 and 75. Information on the distribution of the summary values of scores associated with the acceptance of the disease (AIS) and the quality of life (WHOQoL-BREF), and life satisfaction (SWLS) was presented. For scales AIS and SWLS the distribution of responses following the categorization of score values to the adjective scale was also presented. The distribution of answers was presented in the form of histograms.

An analysis of correlation was used for the determination of the correlation between the acceptance of illness, satisfaction with life and quality of life. Spearman’s non-parametric rank correlation coefficient was used, because of, among others, some asymmetry of the distribution of some scales (especially AIS). To evaluate if the results of the analysis allow the generalization of conclusions regarding the existence of a correlation beyond the study sample, an appropriate statistical test was employed for evaluation if the correlations observed within the sample are an effect of a more general rule for the whole population, or just an accidental result. Statistical tests give the so-called test probability (*p*). Low values of that parameter confirm the statistical significance of the discussed correlation. Selected correlations were illustrated in dispersion graphs.

Evaluation of significance of differences between the compared groups of respondents was completed using the Mann–Whitney test for two groups for the factor of gender, and using the Kruskal-Wallis test for age groups.

Results of analyses of selected traits were illustrated in a box graph including mean values, the typical range of variability and a 95% confidence interval for measuring scales in compared groups. The distribution of all the performed observations was also presented in the form of a dispersion graph superimposed on the box graph.

### Ethical considerations

The study was approved by the Ethical Committee of the Medical University in Białystok, approval no. R-I-002/518/2010. To obtain the approval of the Ethical Committee of the Medical University in Białystok, permission was obtained from the Hospital Director and the Head of the Department where the study was completed. Individual interviews were only started after the purpose of the study had been clearly explained to the participant and an informed consent form was read and signed.

## Results

### Acceptance of illness scale (AIS)

The mean AIS score in the study population was 15.7 with the standard deviation of 4.9, *Me* was 14.0, *c*_25 =_12.0, *c*_75 =_18.0, minimum value = 8, maximum value = 31. The distribution of AIS scores described using selected descriptive statistics is presented in Table 1.

**Table 1:** Distribution of AIS scores described using selected descriptive statistics

**Parameters**	$\overset{―}{x}$	Me	*s*	*c*_25_	*c*_75_	min	max
AIS (points)	15.7	14.0	4.9	12.0	18.0	8	31

**Figure 1:**
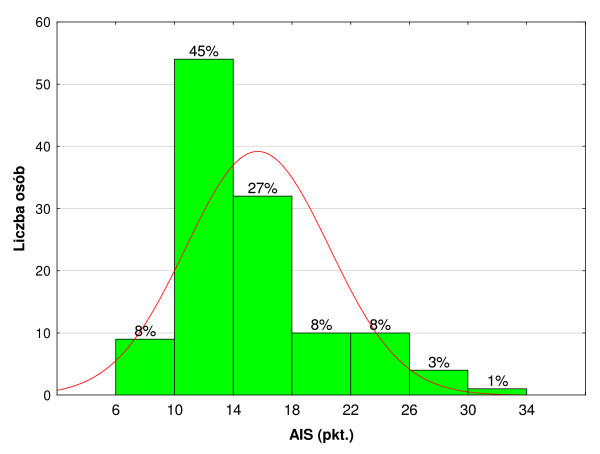
AIS scores.

The majority of people did not accept their condition, which was evident from the relatively low scores in the AIS scale. 45% fit in the range of 10–14 points, and 27% fit in the range of 14–18 points. AIS scores are presented in Figure 1.

A dominating share in the adjective scale fell into the category of “no acceptance”, determined by 71.7% of the respondents, and a “medium” level of acceptance was declared by 25.8% of the participants. Determination of the level of acceptance of illness by respondents using the adjective scale is presented in the Table 2.

**Table 2:** Determination of respondents’ level of acceptance of illness

**Level of acceptance of illness**	**Number**	**Ratio**
No acceptance	86	71.7%
Medium	31	25.8%
High	3	2.5%

### World health organization quality of life – BREF – assessment instrument (WHOQoL-BREF)

**Figure 2:**
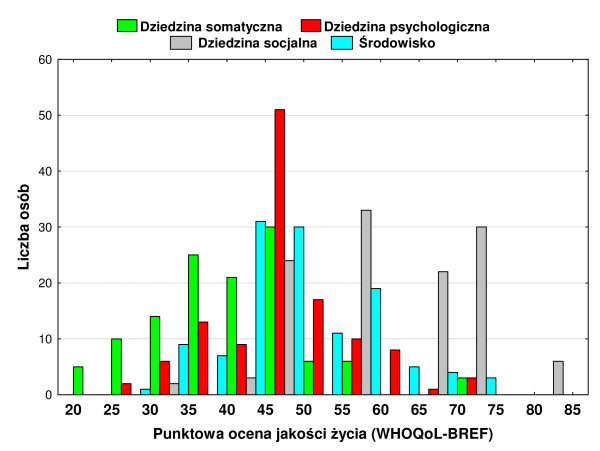
Distribution of values obtained for individual domains in 5-point intervals.

Evaluations of the quality of life in individual domains were re-scaled to values in the range of 0–100. Therefore it was possible to make a comparison between the values obtained from different scales. The mean level of the “somatic domain” was 41.7 with the standard deviation of 10.0, *Me* was 42.9, *c*_25 =_35.7, *c*_75 =_46.4, minimum value = 21.4, maximum value = 75.0. The mean value of the “psychological domain” was 48.8 with the standard deviation of 8.9, *Me* was 50.0, *c*_25 =_43.8, *c*_75 =_54.2, minimum value = 29.2, maximum value = 75.0. The mean level of the “social domain” was 62.8 with the standard deviation of 11.2, *Me* was 58.3, *c*_25 =_58.3, *c*_75 =_75.0, minimum value = 33.3, maximum value = 83.3. A mean level of the “environment” domain was 48.5 with the standard deviation of 9.3, *Me* was 46.9, *c*_25 =_40.6, *c*_75 =_56.3, minimum value = 28.1, maximum value = 71.9. The evaluation of respondents’ quality of life in individual domains is presented in the Table 3. The distribution of values obtained in individual domains in 5-point intervals, with a clear domination of high scores for the social domain, is presented below in a graphic form in Figure 2. The social domain was scored high by respondents in the following score intervals: 45–50, 55–60, 65–70, 70–75, 80–85.

**Table 3:** Evaluation of respondents’ quality of life in individual domains

**Parameters**	$\overset{―}{x}$	Me	*s*	*c*_25_	*c*_75_	min	max
Somatic domain	41.7	42.9	10.0	35.7	46.4	21.4	75.0
Psychological domain	48.8	50.0	8.9	43.8	54.2	29.2	75.0
Social domain	62.8	58.3	11.2	58.3	75.0	33.3	83.3
Environment	48.5	46.9	9.3	40.6	56.3	28.1	71.9

### Satisfaction with life scale (SWLS)

The mean evaluation of satisfaction with life in the SWLS scale is 17.7 points, with the standard deviation of 4.5, *Me* was 18.0, *c*_25 =_15.0, *c*_75 =_21.0, minimum value = 5, maximum value = 28. That means that one in every four persons evaluated their satisfaction with life at a level equal or lower than 15 points. On the other hand, one in every four people evaluated the parameter at the level of at least 21 points. The distribution of SWLS scores described with selected statistics is presented in the Table 4.

**Table 4:** Distribution of SWLS scores described with selected descriptive statistics

**Parameters**	$\overset{―}{x}$	Me	*s*	*c*_25_	*c*_75_	min	max
SWLS	17.7	18.0	4.5	15.0	21.0	5	28

**Figure 3:**
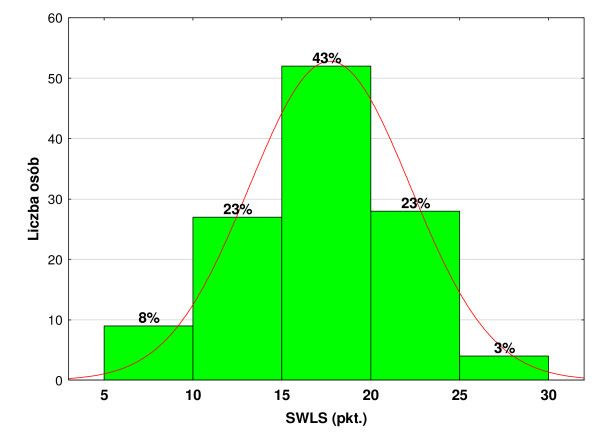
Scores in the SWLS scale.

In this study 23% of the respondents fit into the range of 10–15 points, 43% fit into the range of 15–20 points, and 23% fit into the range of 20–25 points. Scores achieved in the SWLS scale are presented in the Figure 3. A moderate dissatisfaction with life dominates in the study population. People declaring various levels of dissatisfaction constituted almost 70% of the population. The respondents’ determination of their satisfaction with life is presented in the Table 5.

**Table 5:** Respondents’ determination of satisfaction with their life Respondents’ determination of satisfaction with their life

Absolutely dissatisfied	5	4.2%
Very dissatisfied	22	18.3%
Rather dissatisfied	54	45.0%
Neutral	7	5.8%
Rather satisfied	28	23.3%
Very satisfied	4	3.3%

### Correlation between selected scales

This part of the paper is focused on a possible correlation between the acceptance of illness, satisfaction with life and quality of life.

### Acceptance of illness and quality of life and satisfaction with life

Values of Spearman’s rank correlation coefficients, along with the evaluation of the statistical significance of tested correlations between acceptance of illness, satisfaction with life and quality of life are presented in Table 6.

**Table 6:** Correlations between acceptance of illness, satisfaction with life and quality of life

**Satisfaction with and quality of life**		**Acceptance of illness**
		AIS (points)
WHOQoL-BREF	Somatic domain	0.37***
	Psychological domain	0.39***
	Social domain	0.21*
	Environment	0.36***
SWLS		0,40***

The correlation between acceptance of illness and quality of life for the somatic domain was 0.37***. It is a highly statistically significant correlation with low correlation power. The tested correlation between acceptance of illness and quality of life for the psychological domain was 0.39***. It is a highly statistically significant correlation with a low correlation power. Another tested correlation – between acceptance of illness and quality of life for the social domain – was 0.21*. It is a statistically significant correlation with a very low correlation power. The correlation between acceptance of illness and quality of life for the domain of environment was 0.36***. It is a very highly statistically significant correlation with a low correlation power. The correlation between acceptance of illness and satisfaction with life was 0.40***. It is a very highly statistically significant correlation with a low correlation power.

Therefore, a conclusion may be drawn that there is a statistically significant correlation between the level of acceptance of illness and quality of life and satisfaction with life, and a positive sign of the correlation coefficient justifies the statement that the higher the acceptance of illness determines the higher the quality of life. However, the power of the correlation is low, and very low for the correlation of AIS and the evaluation of quality of life in the social domain.

**Figure 4:**
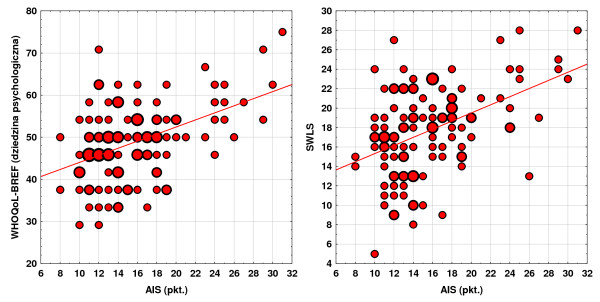
Correlations occurring between acceptance of illness, satisfaction with life and quality of life.

Selected correlations are also presented in dispersion graphs, below. Considering the fact that some combinations of the values of the scales being compared were sometimes repeated in several people, the values of the markers in those graphs is variable, depending on number of represented people. The correlations occurring between the acceptance of illness, satisfaction with life and quality of life are presented in the Figure 4.

### WHOQoL-BREF and SWLS

Additionally, a correlation between quality of life and satisfaction with life was studied. The values of the coefficients of the correlation between satisfaction with life and quality of life are presented in the Table 7. It turns out that the strongest correlation occurred between the satisfaction with life and the evaluation of the quality of life in psychological and environmental domains. However, the power of the correlation is not high. It may be defined, at most, as moderate, which means that SWLS and WHOQoL-BREF scales explore different aspects of satisfaction with life. The studied correlation between satisfaction with life and quality of life for the somatic domain was 0.44*** . It is a very highly statistically significant correlation with a low power of correlation. Another studied correlation – between satisfaction with life and quality of life for the psychological domain – was 0.65***. It is a very highly statistically significant correlation with a moderate power of correlation. On the other hand, the correlation between satisfaction with life and quality of life for the social domain was 0.46***. It is a very highly statistically significant correlation with a low power of correlation; and the correlation between satisfaction with life and quality of life for the domain of environment was 0.60***, which is a very highly statistically significant correlation with a moderate power of correlation.

**Table 7:** Values of coefficients of correlation between satisfaction with life and quality of life

**Satisfaction with life**	**Quality of life (WHOQoL-BREF)**			
	Somatic domain	Psychological domain	Social domain	Environment
SWLS	0.44***	0.65***	0.46***	0.60***

**Figure 5:**
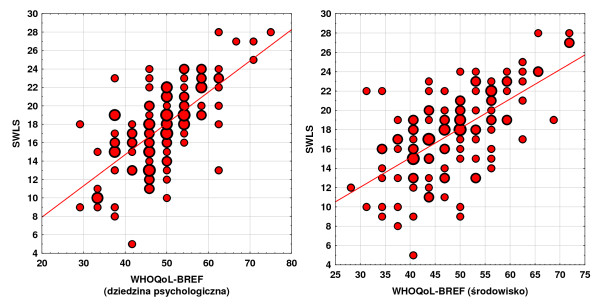
Correlations occurring between satisfaction with life and quality of life in the psychological domain and in the domain of environment.

Selected correlations were also illustrated in dispersion graphs presented below. Correlations occurring between satisfaction with life and quality of life in the psychological domain and in the domain of environment are presented in the Figure 5.

### Correlation of gender and age with quality of life and acceptance of illness

Additionally, a correlation between the quality of the life of women and men with malaria was studied, and the variability of the quality of life in relation to age of surveyed participants. The level of acceptance of illness and satisfaction with life were considered in the same division. A distribution of correlations between the quality of life, acceptance of illness and satisfaction with life, considering the gender of the surveyed people is presented in the Table 8.

**Table 8:** Distribution of correlations between quality of life of studied women and men, considering gender division, presented with selected descriptive statistics

**Measuring scales**	**Gender**				***p***
	**woman**		**man**		
	$\overset{―}{x}$	Me	$\overset{―}{x}$	Me	
AIS (pkt.)	14.7	13.0	16.5	16.0	0.0014**
Somatic domain	41	39	43	43	0.2202
Psychological domain	49	50	49	50	0.7552
Social domain	62	58	64	67	0.3074
Environment	46	44	50	50	0.0073**
SWLS	17.4	17.0	17.9	19.0	0.3999

The mean level (arithmetic mean) of AIS in the study population of women was 14.7, *Me* (median, or middle value, with 50% of measurement results falling below and above that number) was 13.0, and the mean value of AIS in the study population of men was 16.5, *Me* was 16.0, and the test probability was *p* = 0.0014**.

The mean level of quality of life in the domain of environment for women was 46, *Me* was 44, and the mean level of quality of life in the domain of environment for men was 50, *Me* was 50, and the test probability: *p* = 0,0073**.

The above-mentioned results indicate that men tend to demonstrate a higher acceptance of illness. Men also evaluate their quality of life higher in the domain of environment.

Age, however, does not statistically differentiate either acceptance of illness or majority of components of scales WHOQoL-BREF and SWLS significantly. The only correlation was found for a statistically significantly higher (p = 0.0013**) quality of life in the social sphere for relatively older people (Table 9).

**Table 9:** Correlation between scale parameters and age of respondents

**Measuring scales**	**Age**								**p**
	15-18		19-30		31-50		>50		
	$\overset{―}{x}$	Me	$\overset{―}{x}$	Me	$\overset{―}{x}$	Me	$\overset{―}{x}$	Me	
AIS (points)	14.7	14.5	16.5	14.5	14.7	14.0	16.6	16.0	0.2943
Somatic domain	42	43	42	43	40	43	43	39	0.9676
Psychological domain	49	50	49	46	48	50	49	48	0.9276
Social domain	57	54	61	58	67	67	67	67	0.0013**
Environment	45	44	50	50	49	50	47	45	0.2226
SWLS	15.9	17.0	17.9	18.0	17.6	18.0	19.4	19.5	0.1611

**Figure 6:**
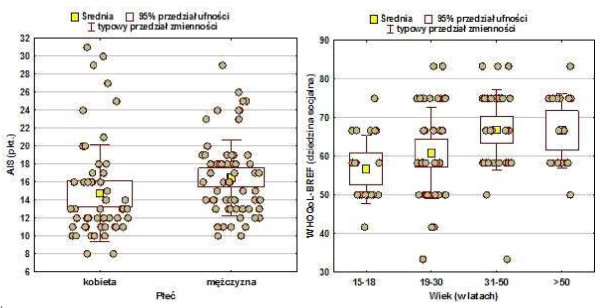
Correlation between parameters of AIS and WHOQoL-BREF scales and respondents’ gender and age.

The results of analyses of individual parameters were illustrated in a box graph, including mean values, a typical variability range and a 95% confidence interval for measuring scales in compared groups. Additionally, the distribution of all observations was presented in the form of a dispersion graph, superimposed on a box graph (Figure 6).

## Discussion

### Acceptance of illness scale (AIS)

The results obtained in this study indicate that the majority of participants did not accept their illness. The fact is confirmed by low scores in the AIS scale. The mean AIS level in the study population was 15.7, with a standard deviation of 4.9. The category of “no acceptance” had a dominating share in the adjective scale. The category was chosen by almost 72% of the respondents. According to literature data, people accepting their illness are those who understand and are aware of the course of the disease, who demonstrate an optimistic and hopeful approach to their condition, show trust in doctors, therapeutic methods, and participate actively in the therapeutic process [13]. Studies on malaria-related knowledge and behavior conducted in various parts of the world – in Africa [14-16], Asia [17] and Europe [18] – reveal a similar, erroneous concept of malaria. Even in Nigeria, Oregba *et al.*, based on a surveyed realized among the population inhabiting the south-western part of the country, found that 65% of respondents had some knowledge on malaria [19].

The people’s ability to submit to medical procedures and the therapeutic process depends on the level of acceptance of those procedures, and on the level of understanding the nature of the disease [20]. Studies by Oladele and Kaun [21] and by other authors [22-25] demonstrated that the knowledge associated with dealing with malaria was significantly correlated with the level of education, and with other important cultural, social and economic factors. Because "in reality, the prevalence of malaria is highest among the poorest members of the society, for those people cannot afford malaria prevention understood as living in homes of a higher standard and in a clean environment. Those people are particularly susceptible to the influence of ineffective diagnostics and therapy” [26].

### World health organization quality of life – BREF – assessment instrument (WHOQoL-BREF)

Our own study demonstrated that the evaluation of the quality of life of malaria patients gave the highest results in the social domain, and the lowest in the somatic (physical) domain. General knowledge is that the main malaria symptom is periodical fever, accompanied by headaches and generalized pain, less common vomiting and diarrhea; and laboratory tests demonstrate thrombocytopenia, reduced WBC count, and anemia in the later stages of the disease [27]. As Nuwaha demonstrated in his studies conducted in Mbarara, in south-western Uganda, the application of traditional medicine and self-therapy may lead to a delay in seeking medical help, or even make it impossible, which leads to increased incidences of malaria, intensification of the disease and an increase in the malaria-related mortality rate [28].

### Satisfaction with life scale (SWLS)

The mean evaluation of satisfaction with life in the SWLS is approximately 18 points. One in every four participants determined his/her satisfaction with life at a level below 15 points. On the other hand, one in every four participants determined his/her satisfaction with life at a level above 21 points. A moderate dissatisfaction with their life dominates in the study population. Nearly 70% of the people are dissatisfied with their life to some extent. Studies by Opiyo *et al.*, conducted in Kenya, demonstrated that an advantage most commonly associated with malaria control was the fact that a family would be happier as nobody would be ill, and there would be the opportunity to save time and money on other “family enterprises” [29].

### Correlations between selected scales

#### Acceptance of illness and quality of life and satisfaction with life

It is reasonable to expect that individuals demonstrating a higher acceptance of illness will also declare a higher satisfaction with life and quality of life, just as it was demonstrated in the study by Lewko et al. conducted on 59 patients with type I and II diabetes [13]. Of course, the direction of that correlation is a disputable question, for it is equally justified to state that a higher quality of life leads to a higher acceptance of illness, and vice versa. From a statistical point of view, that problem is irrelevant, because both compared parameters are treated symmetrically in the analysis of correlation. The studies demonstrated that there is a statistically significant correlation between the level of acceptance of illness and quality of life and satisfaction with life. Therefore, an assumption may be made that some correlation also exists beyond the study population, in the target population; and a positive sign of the correlation coefficient justifies the statement that the higher the acceptance of illness determines a higher quality of life, so the direction of the correlation is logical and consistent with common sense expectations; and that the power of the correlation is low, which means that not only an attitude towards the disease determines the quality of life of malaria patients.

### WHOQoL-BREF and satisfaction with life scale (SWLS)

The results obtained demonstrated that the strongest correlation occurred between satisfaction with life and the evaluation of quality of life in the psychological domain and in the domain of the environment. However, the power of the correlation is not high, and can be at most assessed as moderate, which means that SWLS and WHOQoL-BREF scales test different aspects of satisfaction with life. Studies by Ojakaa *et al.,* conducted in two malaria-endemic regions of Kenya, demonstrated a commonly accepted view that malaria has a negative effect on the economic status and stability of a family [30].

### Correlation of gender and age with quality of life and acceptance of illness

An analysis of the results obtained demonstrated that men are characterized by a higher acceptance of illness. Men also evaluate their quality of life in the environmental domain higher. There is no statistically significant age-related difference in the acceptance of illness and the majority of components of WHOQoL-BREF and SWLS scales. Studies by Opiyo *et al.* conducted among people inhabiting the Rusinga Island in Kenya demonstrated that age and the level of education constitute the main factors deciding on a good knowledge of malaria and appropriate malaria-related behavior [29]. Results of the study by Ojakaa *et al.* conducted in two malaria-endemic regions of Kenya demonstrated that decisions on the vaccination of children are made in a variable manner [30].

On the other hand, studies completed in Nigeria by Iwalokun *et al.* demonstrated that the use of western medicines was associated with a formal education and younger age, while self-medication was usually practiced by the men [31]. According to Nsagha’s just observation presented in his studies, in Africa, as elsewhere, women are responsible for the nursing and health care of children. Women are therefore more likely to seek and use anti-malarial treatment [32].

## Conclusions

Empirical material collected during the studies, its statistical elaboration and interpretation of results define the scope of the final conclusions. The study demonstrated that the majority of people do not accept their illness, that the evaluation of quality of life was the highest in the social domain and the lowest in the somatic domain. Moreover, the study demonstrated the existence of a statistically significant correlation between the level of acceptance of illness and quality of life and satisfaction with life. The strongest correlation exists between satisfaction with life and the evaluation of quality of life in psychological and environmental domains. The results of the study indicate that men evaluate their quality of life higher in the environmental domain and demonstrate a higher acceptance of illness, and also that there is a correlation regarding a significantly higher quality of life in the social domain in relatively older people. It may be also concluded that there is a statistically significant correlation between the level of acceptance of illness and quality of life and satisfaction with life. Therefore, an assumption may be made that some correlation also exists beyond the study population, in the target population. A conclusion may also be drawn that a higher acceptance of illness determines a higher quality of life.

## Abbreviations

AIS; Acceptance of Illness Scale; SWLS, Satisfaction with Life Scale; WHOQoL-BREF, World Heatlh Organization Quality of Life; BREF, p – test probability also called a critical significance level; $\overset{―}{x}$, Arithmetic mean; Me, Median, middle value; max, Maximum value; min, Minimum value; s, Standard deviation; c25, Centile 25; c75, Centile 75.

## Conflict of interests

The authors declare no conflict of interests.

## Authors’ contributions

All the authors were involved in the design of the study. KVDO was responsible for the implementation of the study in the field and the draft of the manuscript. EKK conceived the study, and participated in its design and coordination and supported the drafting of the manuscript. ER coded the data and supervised data entry. WLN and RO analyzed the study data. All the authors were involved in finalizing the manuscript, read and approved the final version.
